# Supplementary material for: Selective inhibition of TGF-β1 produced by GARP-expressing Tregs overcomes resistance to PD-1/PD-L1 blockade in cancer
Source: Nat Commun. 2020 Sep 11;11:4545. doi: 10.1038/s41467-020-17811-3 (PMC7486376; doi:10.1038/s41467-020-17811-3)
Supplement: Supplementary file 1 — Supplementary Information [file 41467_2020_17811_MOESM1_ESM.pdf]

Supplementary Information

Selective inhibition of TGF-β1 produced by GARP-expressing Tregs overcomes resistance to PD-1/PD-L1 blockade in cancer

Authors: Grégoire de Streel, Charlotte Bertrand, Nicolas Chalon, Stéphanie Liénart, Orian Bricard, Sara Lecomte, Julien Devreux, Mélanie Gaignage, Gitte De Boeck, Lore Mariën, Inge Van De Walle, Bas van der Woning, Michael Saunders, Hans de Haard, Elien Vermeersch, Wim Maes, Hans Deckmyn, Pierre G. Coulie, Nicolas van Baren, Sophie Lucas

	anti-GARP:TGF-β1 Clone 58A2		Isotype control Motavisumab	
	mIgG2a WT	mIgG2a FcD	mIgG2a WT	mIgG2a FcD
**FcγRI**	**525**	*nb*	**533**	*nb*
**FcγRII**	**46**	*nb*	**91**	*nb*
**FcγRIII**	*nb*	*nb*	**16**	*nb*
**FcγRIV**	**227**	*nb*	**199**	*nb*

Supplementary Figure 1 Binding of anti-GARP:TGF-β1 and isotype control mAbs to mouse FcγRs
Biotinylated recombinant extracellular domains of the indicated mouse FcγRs were immobilized to a sensor chip and binding by anti-GARP:TGF-β1 clone 58A2 or a control antibody (Motavisumab), in WT or FcD mIgG2a formats, was measured using Surface Plasmon Resonance (Biacore). Due to very fast dissociation, kinetic constants could not be determined. Values indicate binding response units (RU). *nb*: no binding.

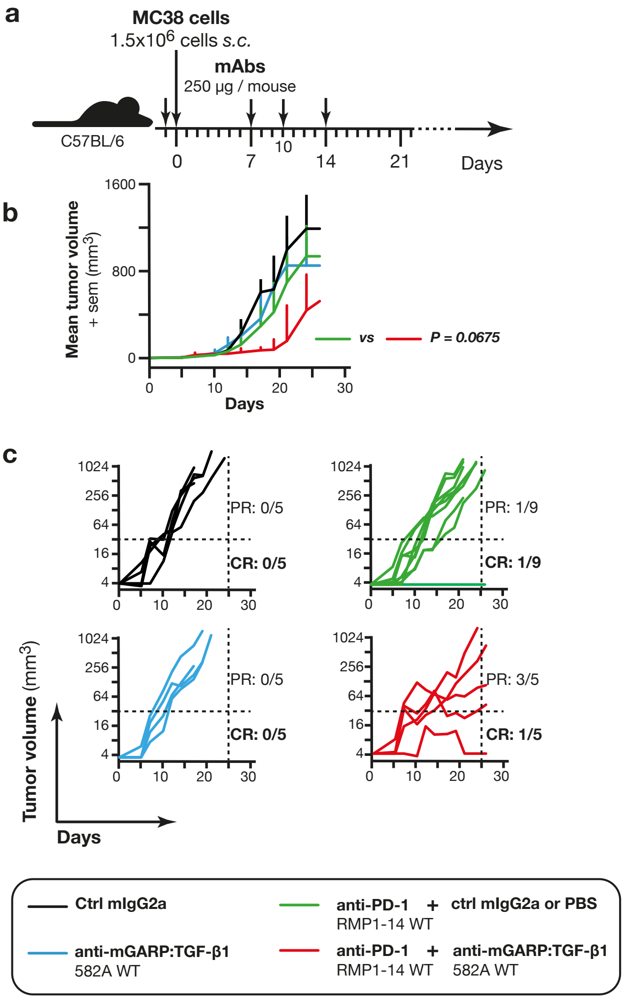

Supplementary Figure 2 Combined blockade of GARP:TGF-β1 and PD-1 shows anti-tumor efficacy against MC38 tumors in WT C57BL/6 mice
a Experimental design. C57BL/6 mice were injected *s.c.* with live MC38 cells on day 0. Tumor diameters were measured two to three times a week. mAbs were injected *i.p.* on days -1, 7, 10 and 14. Mice treated with anti-PD-1 alone or anti-PD-1 combined with an isotype control (mIgG2a) were pooled together (n=9). Mice were sacrificed when the tumor surface was ≥ 200 mm^2. **b, c** Evolution of mean (+ s.e.m) and individual tumor volumes in the treatment groups. Ratios indicate the proportions of Complete Responders (CR, *i.e.* live mice with no detectable tumor at the end of the experiment) and Partial Responders (PR, *i.e.* mice alive on day 25 that carry a tumor >32 mm^3 at the time of euthanasia). Dotted horizontal and vertical lines indicate the two arbitrary limits (tumor volume of 32 mm^3 and day 25) used to identify PR and CR. *P* value for the indicated relevant comparison was calculated with a mixed effects model, as recommended for analyses of longitudinal data (Liu et al., 2010; Sugar et al., 2012), with a *post-hoc* Tukey's test for multiple comparisons.

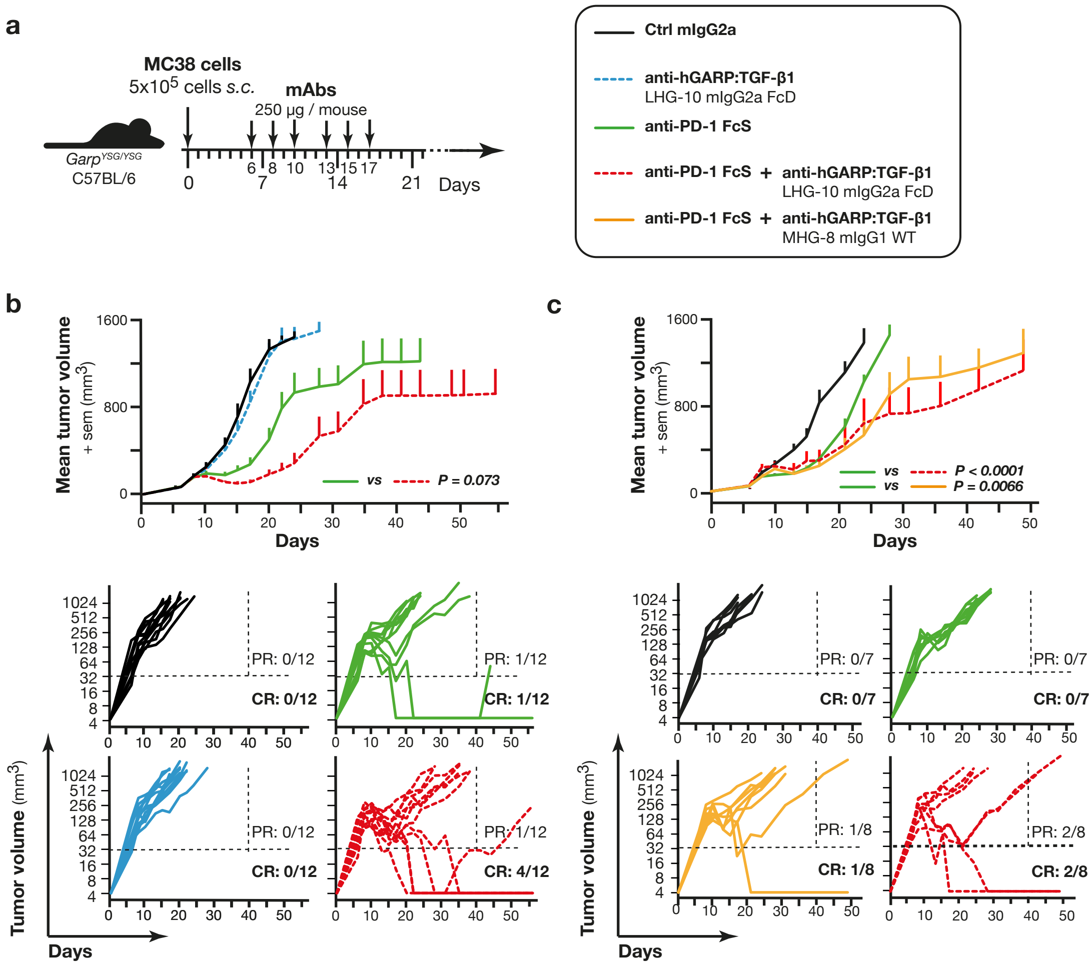

Supplementary Figure 3 Combined blockade of GARP:TGF-β1 and PD1 shows anti-tumor efficacy against MC38 tumors in *Garp*$^{YSG/YSG}$ C57BL/6 mice

a Schematic representation of the experimental design. *Garp*$^{YSG/YSG}$ C57BL/6 mice were injected *s.c.* with live MC38 cells on day 0. Tumor diameters were measured twice a week. On day 6, mice were randomized in the indicated experimental groups, and mAbs were injected *i.p.* every 2-3 days from day 6 to 17. Mice were sacrificed when the tumor surface was ≥ 200 mm^2. **b, c** Evolution of mean and individual tumor volumes in various the treatment groups (b: n=11-12 mice per group; c: n=7-8 mice per group). Ratios indicate the proportions of CR (mice alive at the end of the experiment with no detectable tumor) and PR, (mice alive on day 40 that carry a tumor >32 mm^3 at the time of euthanasia). Dotted horizontal and vertical lines indicate the two arbitrary limits (tumor volume of 32 mm^3 and day 40) used to identify PR and CR. *P* values for relevant comparisons are indicated and were calculated with a mixed effects model (Liu et al., 2010; Sugar et al., 2012), with a *post-hoc* Tukey's test for multiple comparisons.

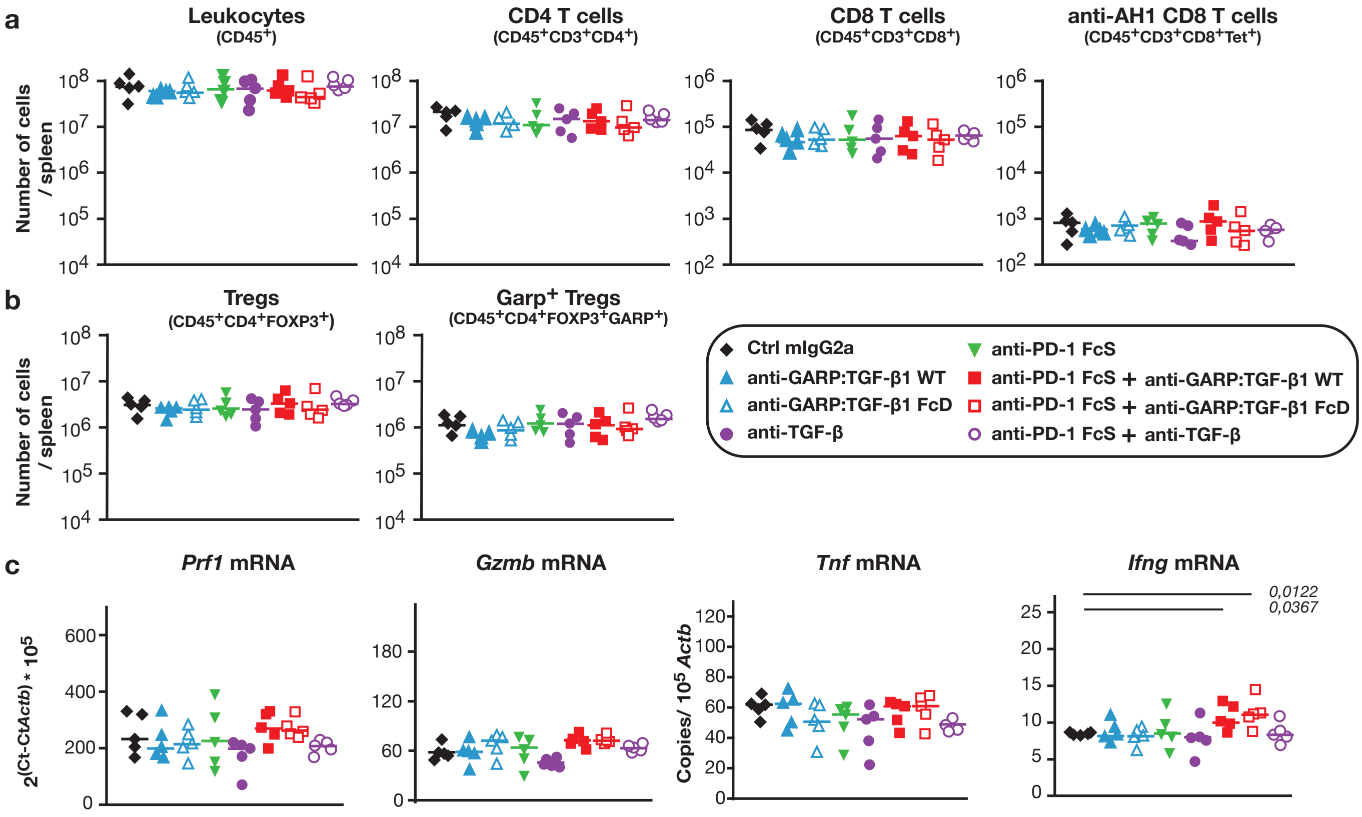

Supplementary Figure 4 Analyses of spleens collected from mice shown in Fig. 5.

a, b Numbers of various subsets of cells as determined by flow cytometry. Each data point represents the value measured in one mouse (n=5 mice/-group). Horizontal bars represent medians per group. **c** Expression of genes encoding T cell-effector molecules, as determined by RT-qPCR. Data points and horizontal bars as in a. Numbers in italics indicate *P* values < 0.05 by comparison to the control group (isotype mIgG2a), calculated with a two-sided Wilcoxon test. Results shown here are representative of at least 3 independent experiments.

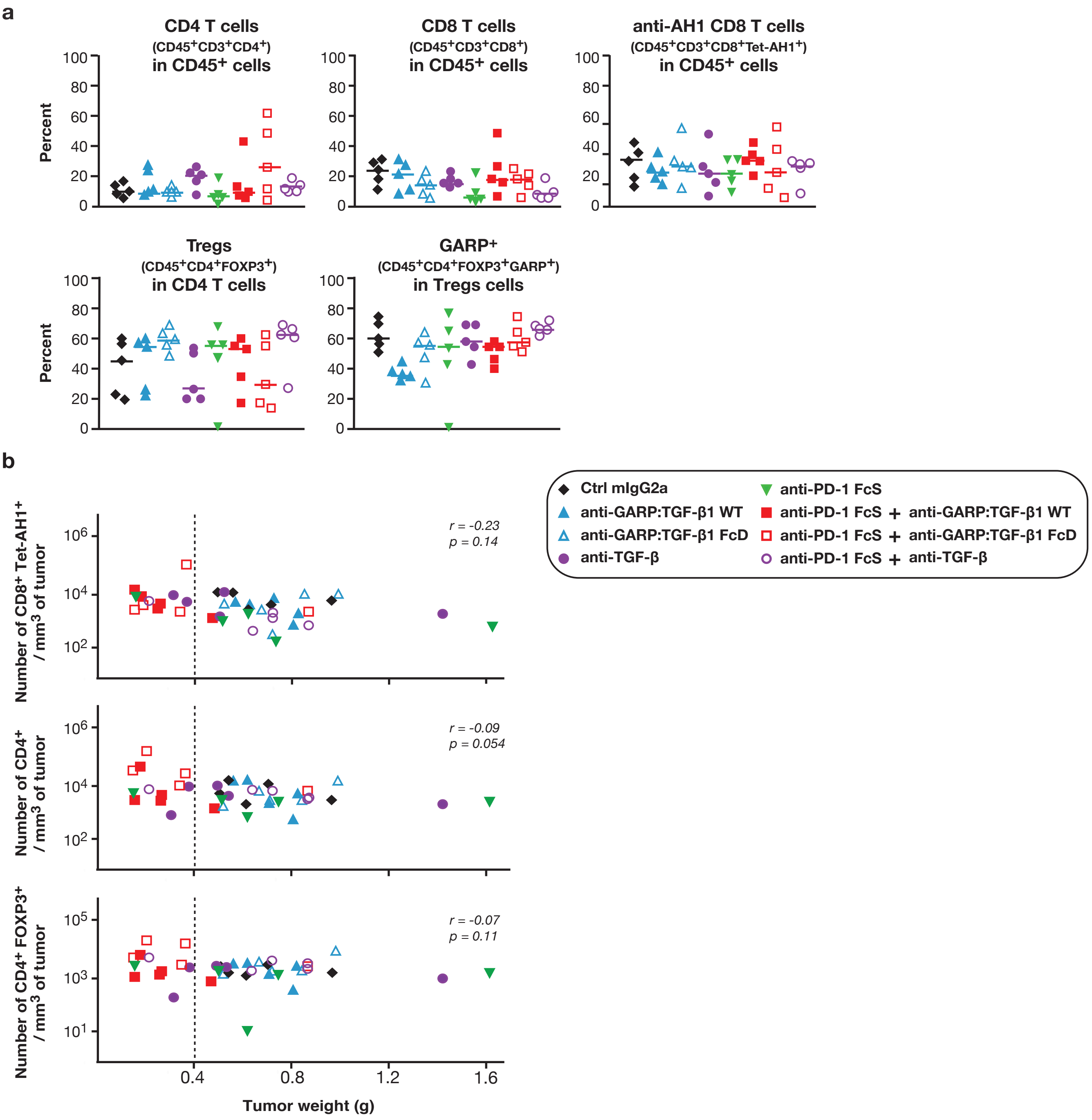

Supplementary Figure 5 Combined blockade of GARP:TGF-β1 and PD-1 does not modify numbers of cells or proportions of leukocyte subsets infiltrating CT26 tumors

a Proportion of various subsets of immune cell infiltrating the tumor**.** Each data point represents the value measured in one mouse, and horizontal bars the median per group (n=5 mice/group). **b** Correlation between numbers of various subsets of cells infiltrating one mm^3 of tumor with tumor weight in the corresponding mouse at day 13. r = Pearson's correlation coefficient, and corresponding p value as calculated with an F-test. Doted vertical bars indicate an arbitrary limit set to define smallest tumors as in Fig. 5 (n=5 mice/group).

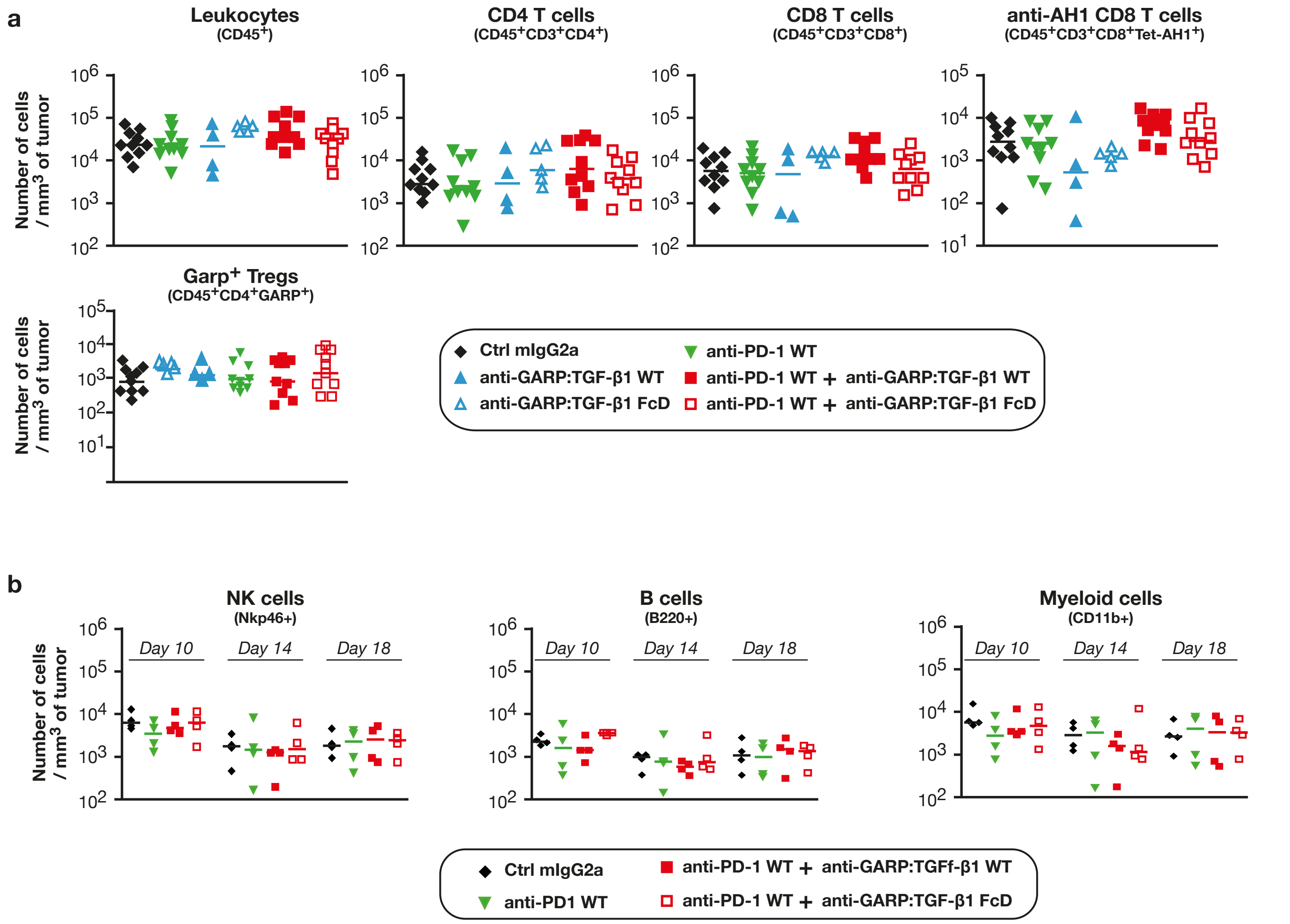

Supplementary Figure 6 Combined blockade of GARP:TGF-β1 and PD-1 does not modify numbers of cells infiltrating CT26 tumors
Independent experiments similar to that shown in Fig. 5.
a Pooled of two independent experiments showing numbers of various subsets of cells infiltrating one mm^3 of tumor, as determined by flow cytometry. Each data point represents the value measured in one mouse, and horizontal bars the median per group. **b** BALB/c mice were injected *s.c.* with live CT26 tumor cells on day 0 and treated *i.p.* with the indicated mAbs on days 6, 10 and 14. Mice were sacrificied on days 10, 14 or 18 (n= 4 mice per group and per time point) to collect tumors for flow cytometry analyses. Graphs represent numbers of various subsets of cells infiltrating one mm^3 of tumor. Each data point represents the value measured in one mouse, and horizontal bars medians per group.

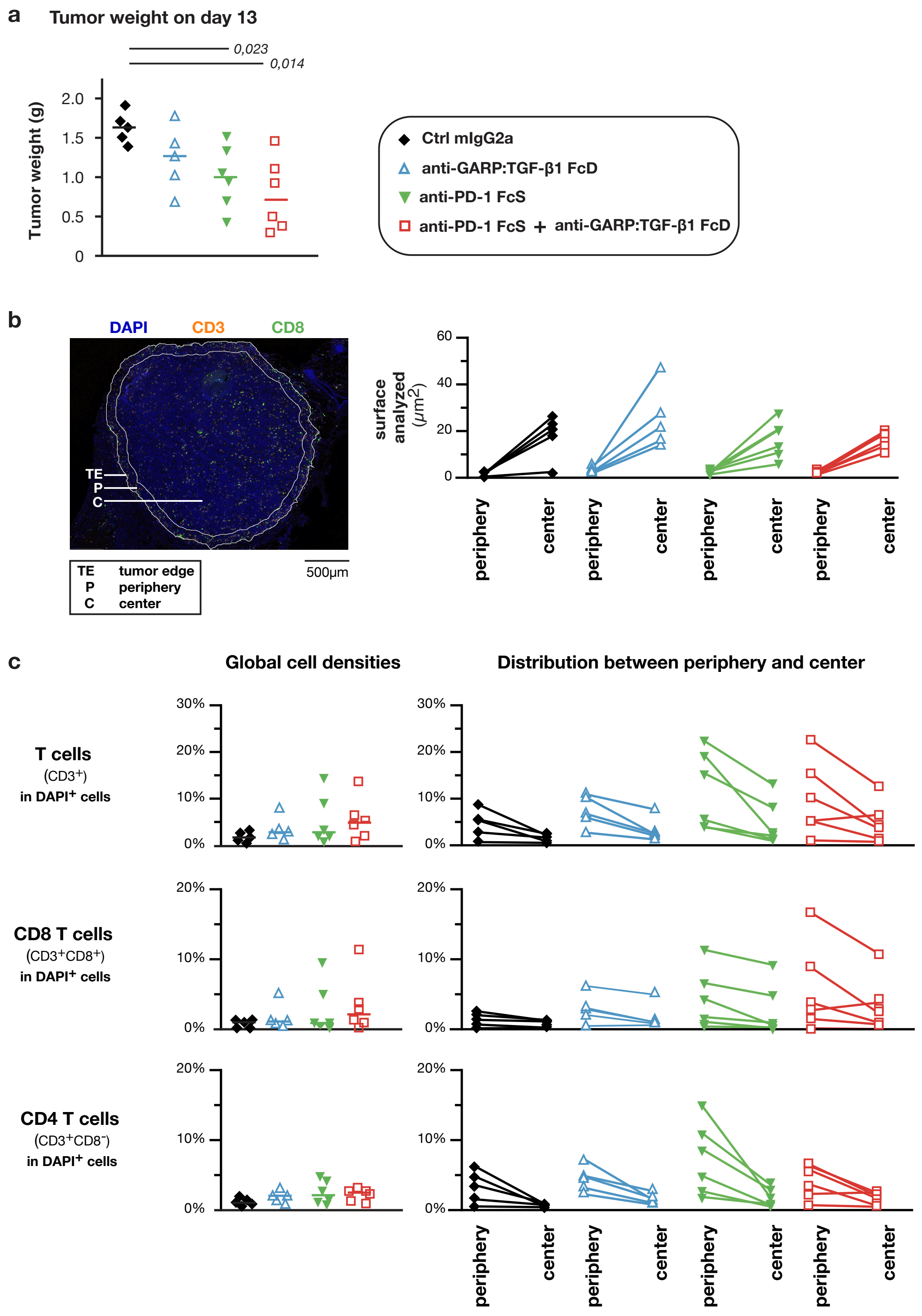

Supplementary Figure 7 Combined blockade of GARP:TGF-β1 and PD-1 does not increase T cells infiltration and migration towards the tumor center by comparison to anti-PD-1 alone

BALB/c mice (n=5-6/group) were injected with CT26 cells on day 0 and treated with mAbs on days 6, 9 and 12, as illustrated in Fig 2. Tumors were collected on day 13. **a** Weight of tumors on the day of collection. Each data point represents the value in one mouse, and horizontal bars the median per group. Numbers in italics show *P* values <0.05 for the comparisons with the control group (isotype ctrl mIgG2a), as calculated with a two-sided Wilcoxon test. **b** Left: Representation of zones defined for analysis of T cell densities (one representative tumor section is shown). Periphery is comprised within tumor edge and a concentric line located at 100µm from tumor edge. Center is defined as the rest of the tumor. Right: Surface of peripheral and central zones analysed in one tumor section per mouse. Data points from one mouse are connected by a straight line. **c** Densities of total T, CD8 T and CD4 T cells in DAPI^{+} cells in the entire section (left) or in periphery and center (right), as determined by immunofluorescence microscopy followed by digital image analysis and counting. Data points and horizontal bars as in a and b. No *P* value <0.05.

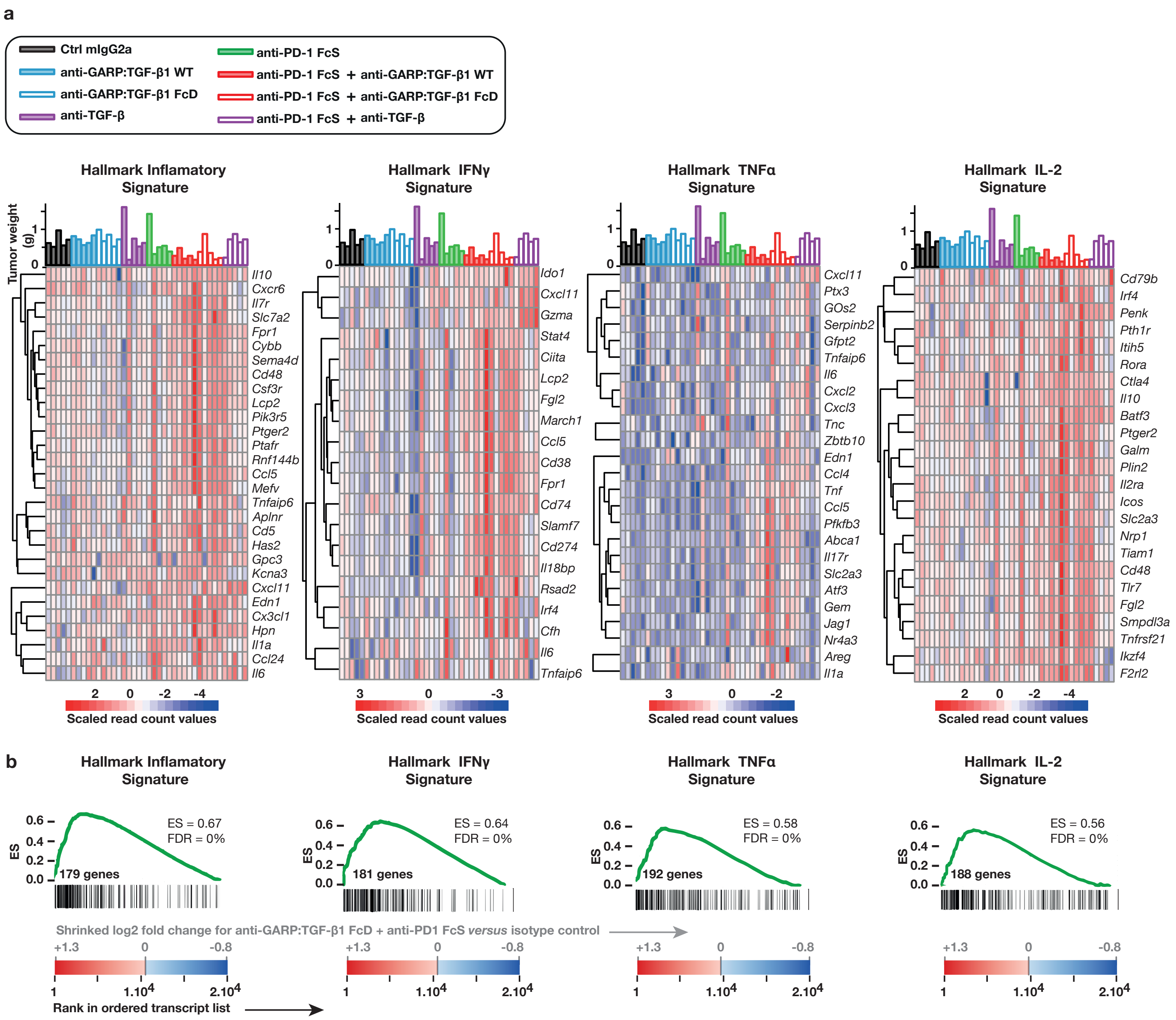

Supplementary Figure 8 Signatures for responses to inflammatory and T cell derived cytokines are enriched in tumors from mice treated with anti-GARP:TGF-β1 combined with anti-PD-1 mAbs

a Bar graphs show weight of tumors collected on day 13 in individual mice from experiment in Fig. 5. Heat maps show scaled transcript read counts in the corresponding mice as determined by RNAseq. Transcripts indicated in each heat map correspond to genes represented by larger dots in the violin plots of Fig. 5e (*i.e.* genes from the indicated hallmark signatures that show a fold change ≥2 for the ratio between mean expression in the treated group versus the isotype control group). **b** GSEA plots for the comparisons between groups treated with the anti-GARP:TGF-β1 FcD + anti-PD-1 FcS combination and isotype control. Transcripts identified by RNAseq are ordered by shrinked log2 fold change between the two groups. Enrichment in the ordered lists are plotted as green curves. Enrichment score (ES) and false discovery rate (FDR) statistics are indicated for each signature. FDR was obtained by calculating ES for 1000 gene-set permutations.

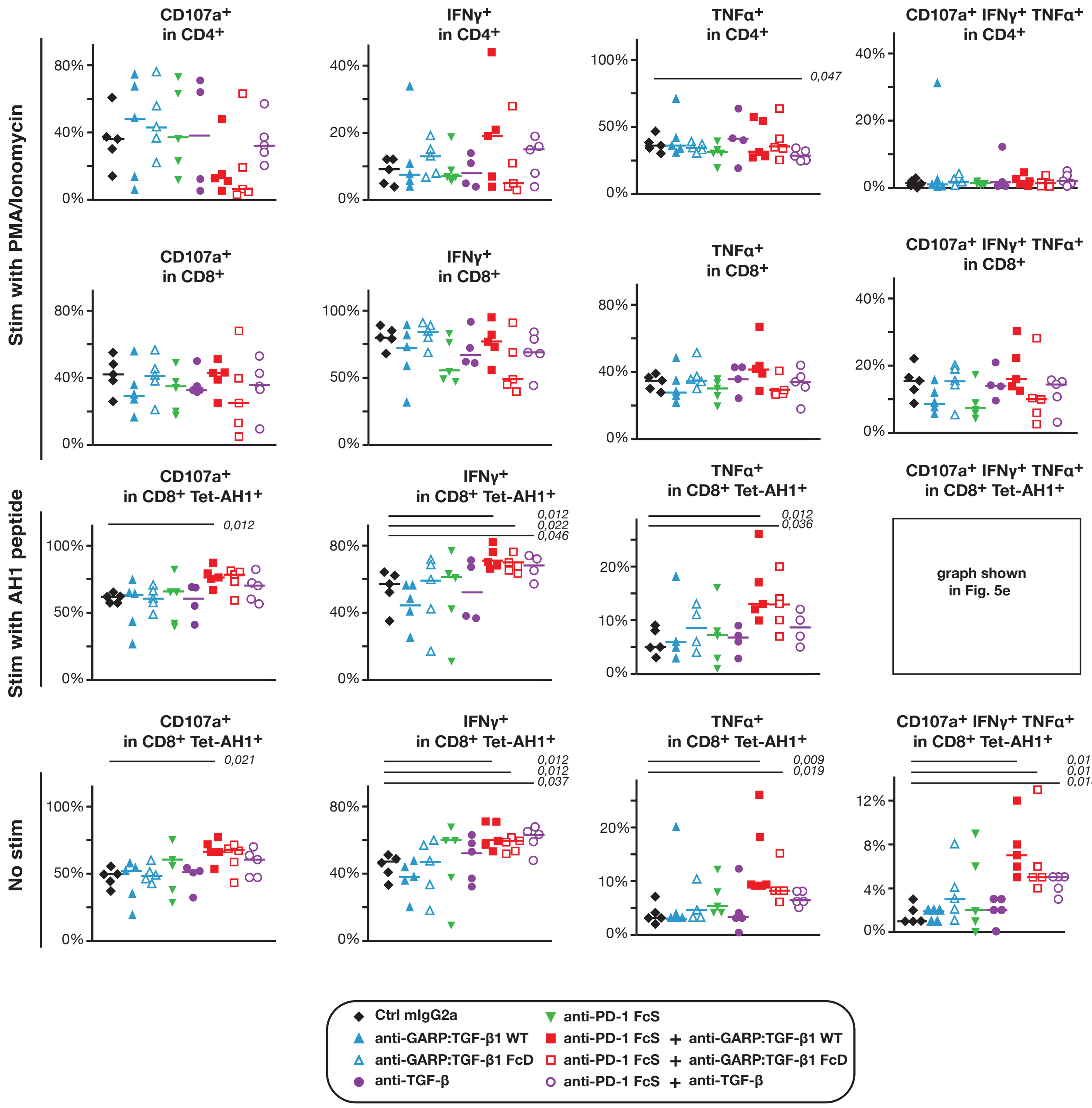

Supplementary Figure 9 Combined blockade of GARP:TGF-β1 and PD-1 increases effector functions of anti-tumor CD8+ T cells, but not that of other T cells infiltrating CT26 tumors
Cells collected from tumor samples shown in Fig. 5 were left resting or stimulated during 4 hours in vitro with PMA/Ionomycin or the AH1 peptide, then stained against various surface markers, including surface CD107a, and intracellular IFNγ and TNFα, prior to flow cytometry. Graphs show the proportions of cells expressing the indicated markers within CD4+, CD8+ or CD8+ Tet-AH1+ gates. Each data point represents the value in one mouse. Horizontal bars represent medians per group. Numbers in italics show *P* values <0.05 for the comparisons with the control group (isotype ctrl mIgG2a), as calculated with a two-sided Wilcoxon test. Results shown here are representative of at least 3 independent experiments (n=4-5 mice/group).

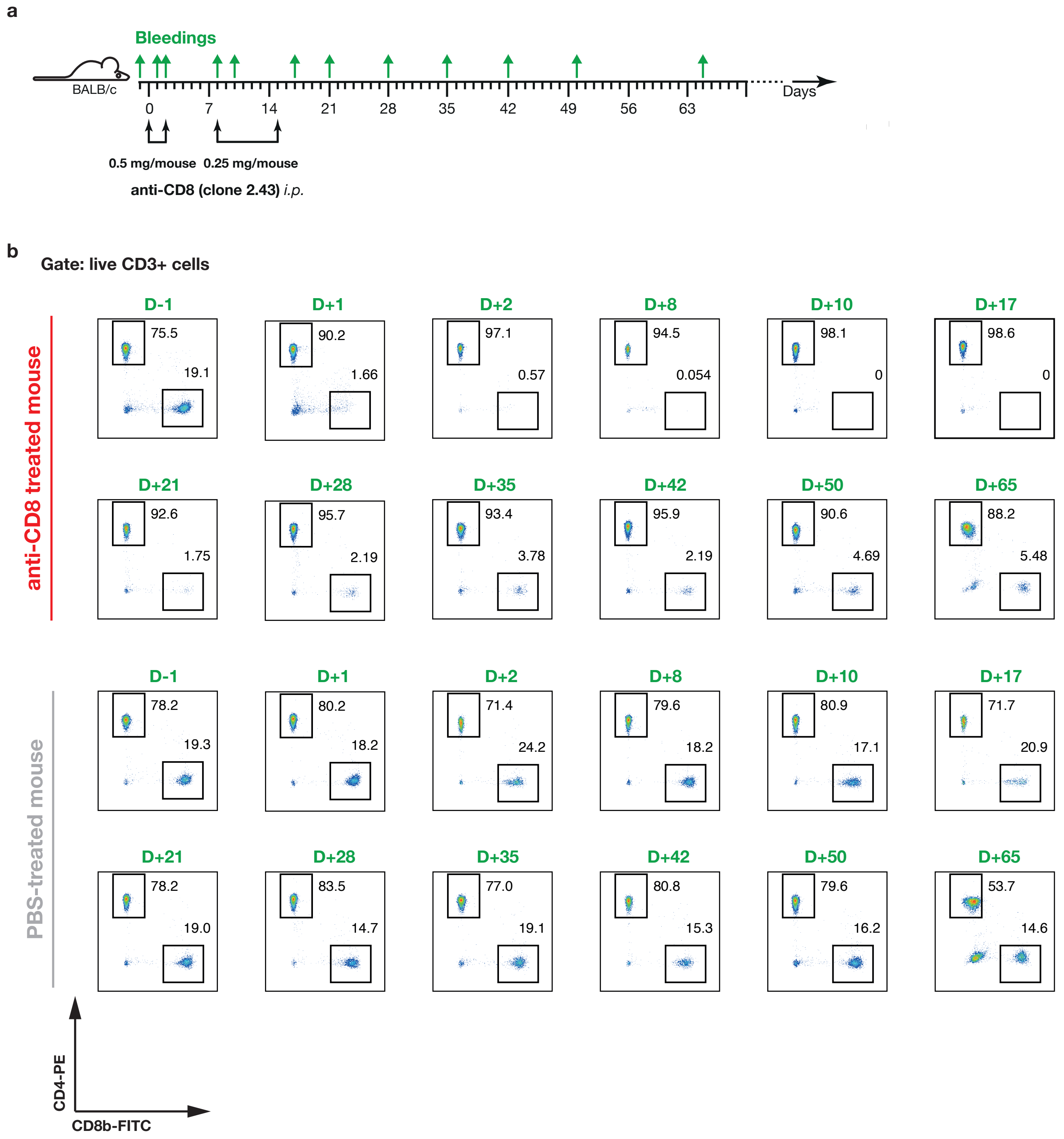

Supplementary Figure 10 Anti-CD8 mAb depletes CD8+ T cells in BALB/c mice
a Experimental design. Balb/c mice received *i.p* injections of depleting anti-CD8 mAb clone 2.43 (n=1) or PBS as control (n=1), as indicated on the scheme, and were bled at various time points to measure proportions of CD8 T cells in the blood by flow cytometry. **b** Results of flow cytometry analyses. Density plots are gated on live CD3+ cells. Values indicate percentage of CD3+ cells present in the illustrated rectangular gates (CD4+ or CD8+ cells in blood CD3+ cells), anti-CD4-PE (clone RM4-4, Biolegend) and anti-CD8b (clone H35-17.2, eBioscience) which has been verified to not compete with depleting antibody.
D: day.

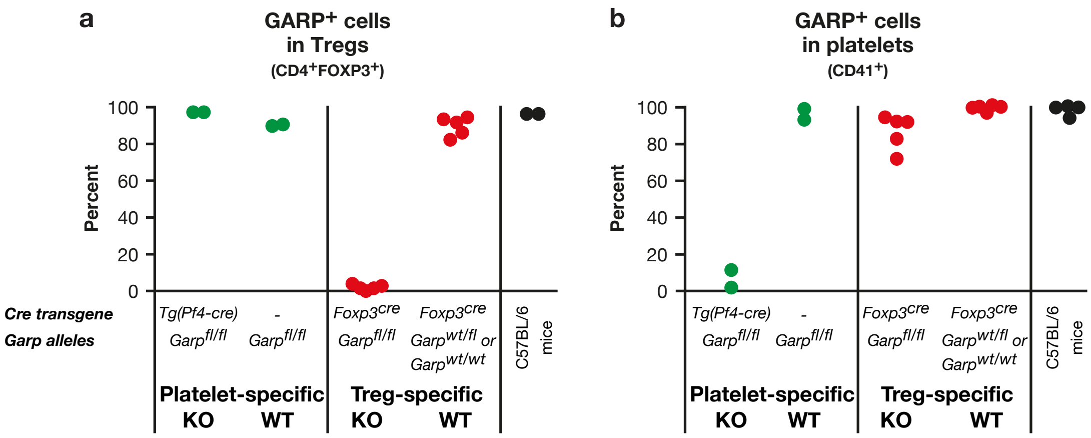

Supplementary Figure 11 Cell type-specific loss of GARP expression in Treg- and platelet- specific *Garp* KO mice.
a Splenocytes from mice of the indicated genotypes were isolated and stimulated *in vitro* with anti-CD3/28 coated beads during 24 hours, then analysed by flow cytometry. Each data point indicates proportions of GARP positive cells in $CD4^{+}FOXP3^{+}$ cells (Tregs) for one mouse (n = 2 to 5). **b** Platelets from mice of the indicated genotypes were isolated from fresh blood and analysed by flow cytometry. Each data point indicates proportions of GARP positive cells in $CD41^{+}$ cells (platelets) from one mouse (n = 2 to 5).

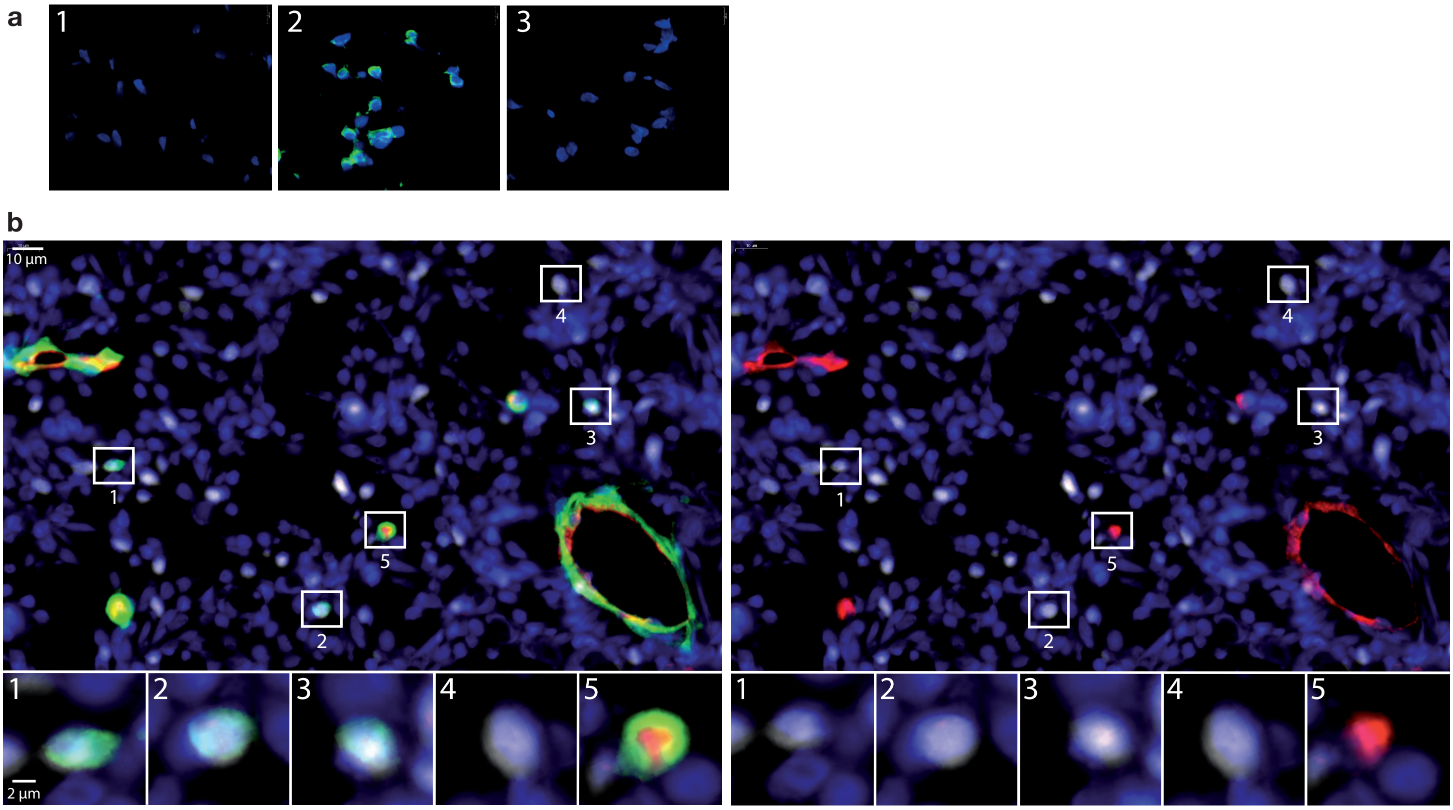

Supplementary Figure 12 Multiplexed IF allows to identify GARP-expressing Tregs and endothelial cells in sections of frozen human tonsils
a Immunofluorescence staining with the anti-GARP MHG-6 mouse monoclonal antibody on sections of frozen cell pellets from cultured Jurkat cells (1) or Jurkat transfected with a GARP-encoding plasmid (2); GARP appears as a green signal, nuclei in blue; 3: same as 2 without MHG-6. **b** Left panel: mIF staining of GARP (green), FOXP3 (white) and CD34 (endothelial cells, red) on a frozen tonsil section. Merged green and red channels appear in yellow. Bottom panels: enlarged view of three FOXP3$^+$GARP$^+$ cells (1-3), a FOXP3$^+$GARP$^-$ cell (4) and a GARP$^+$CD34$^+$ capillary vessel (5). Right panel: idem without the GARP (green) channel. Similar results were obtained with two other anti-GARP antibodies (clones Plato-1 and LHG-10).

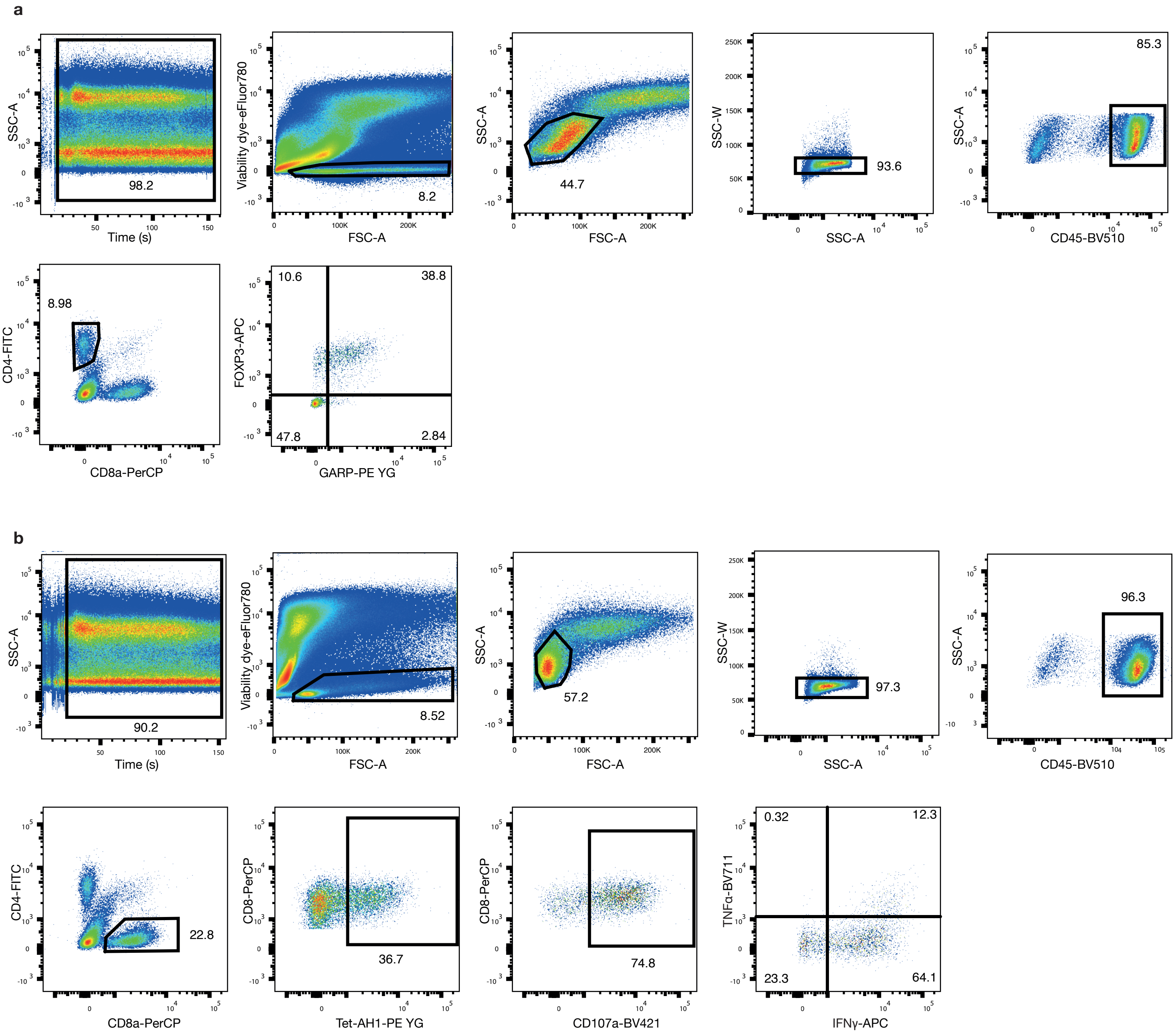

Supplementary Figure 13 Examples of gating strategies used for flow cytometry analyses shown in Fig. 5 and Supplementary Figures 5-6

a Representative example of the gating strategy applied to evaluate the number of Tregs and GARP$^+$ Tregs infiltrating the tumor. **b** Same to evaluate infiltration of CD8$^+$ Tet-AH1$^+$ CD107a$^+$ IFNγ$^+$ TNFα$^+$ cells

a

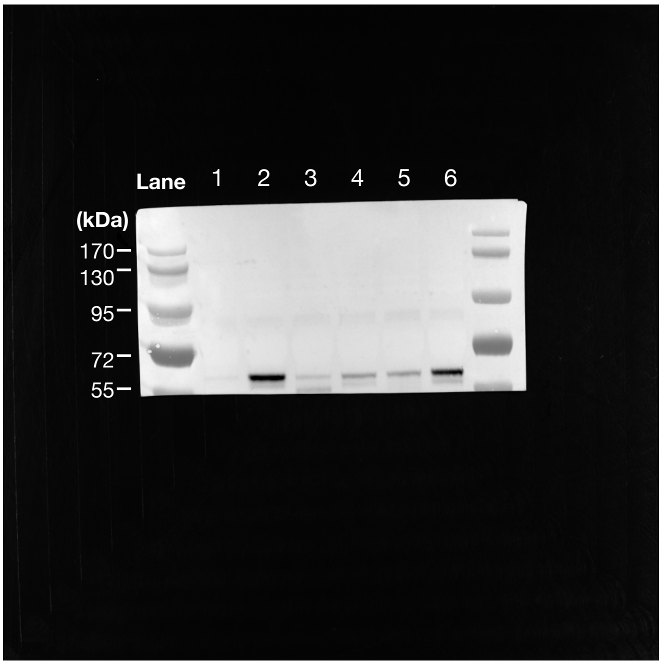

b

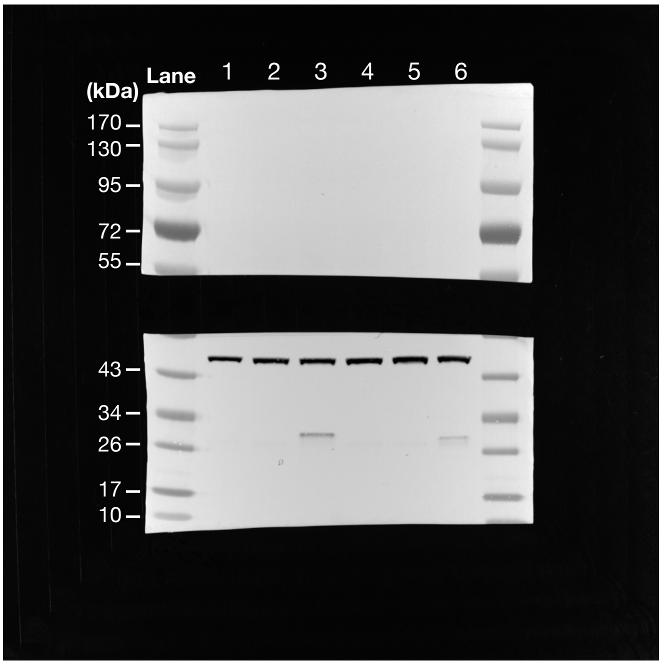

Supplementary Figure 14. Full scans of Western Blot images shown in Fig.1.
a. pSMAD2
b. mouse Actin

Supplementary Table 1. Primers sequences

Sequence	Definition
TCAAGTGGCATAGATGTGGAAGAA	Sense primer in *mIfng* mRNA
TGGCTCTGCAGGATTTTCATG	Antisense primer in *mIfng* mRNA
TCACCATCCTTTTGCCAGTTCCTCCAG	Taqman probe in *mIfng* mRNA
AAGAGAGCAAGGACAACACTC	Sense primer in *mGZMb* mRNA
CATGTCCCCCGATGATCTC	Antisense primer in *mGZMb* mRNA
ACAAGGTCAGCAGTAGCAGGAGGA	Taqman probe in *mGZMb* mRNA
CAGTAGAGTGTCGCATGTACAG	Sense primer in *mPrf1* mRNA
GATGAGCCTGTGGTAAGCAT	Antisense primer *in mPrf1* mRNA
TCGCCTGGTACAAAAACCTCCACTC	Taqman probe in *mPrf1* mRNA
CATCTTCTCAAAATTCGAGTGACAA	Sense primer in *mTNFa* mRNA
GGGTTGTACCTTGTCTACTCCCA	Antisense primer in *mTNFa* mRNA
CACGTCGTAGCAAACCACCAAGTGGA	Taqman probe in *mTNFa* mRNA
