# Supplementary material for: Selective inhibition of TGF-β1 produced by GARP-expressing Tregs overcomes resistance to PD-1/PD-L1 blockade in cancer
Source: Nat Commun. 2020 Sep 11;11:4545. doi: 10.1038/s41467-020-17811-3 (PMC7486376; doi:10.1038/s41467-020-17811-3)
Supplement: Supplementary file 3 — Description of Additional Supplementary Files [file 41467_2020_17811_MOESM3_ESM.pdf]

Description of Additional Supplementary Files

File Name: Supplementary Data 1
Description: Ordered transcript lists used for GSEA analyses in figure 8.

File Name: Supplementary Data 2
Description: Experimental gene sets used for GSEA analyses in figure 8.
