# Supplementary material for: Selective inhibition of TGF-β1 produced by GARP-expressing Tregs overcomes resistance to PD-1/PD-L1 blockade in cancer
Source: Nat Commun. 2020 Sep 11;11:4545. doi: 10.1038/s41467-020-17811-3 (PMC7486376; doi:10.1038/s41467-020-17811-3)
Supplement: Supplementary file 6 — Reporting Summary [file 41467_2020_17811_MOESM6_ESM.pdf]

nature research

Corresponding author(s): Sophie Lucas

Last updated by author(s): 26/06/2020

Reporting Summary

Nature Research wishes to improve the reproducibility of the work that we publish. This form provides structure for consistency and transparency in reporting. For further information on Nature Research policies, see Authors & Referees and the Editorial Policy Checklist .

Statistics

For all statistical analyses, confirm that the following items are present in the figure legend, table legend, main text, or Methods section.

n/a	Confirmed	
☐	☒	The exact sample size (*n*) for each experimental group/condition, given as a discrete number and unit of measurement
☐	☒	A statement on whether measurements were taken from distinct samples or whether the same sample was measured repeatedly
☐	☒	The statistical test(s) used AND whether they are one- or two-sided<br>*Only common tests should be described solely by name; describe more complex techniques in the Methods section.*
☒	☐	A description of all covariates tested
☐	☒	A description of any assumptions or corrections, such as tests of normality and adjustment for multiple comparisons
☐	☒	A full description of the statistical parameters including central tendency (e.g. means) or other basic estimates (e.g. regression coefficient) AND variation (e.g. standard deviation) or associated estimates of uncertainty (e.g. confidence intervals)
☐	☒	For null hypothesis testing, the test statistic (e.g. *F*, *t*, *r*) with confidence intervals, effect sizes, degrees of freedom and *P* value noted<br>*Give P values as exact values whenever suitable.*
☒	☐	For Bayesian analysis, information on the choice of priors and Markov chain Monte Carlo settings
☒	☐	For hierarchical and complex designs, identification of the appropriate level for tests and full reporting of outcomes
☐	☒	Estimates of effect sizes (e.g. Cohen's *d*, Pearson's *r*), indicating how they were calculated

Our web collection on statistics for biologists contains articles on many of the points above.

Software and code

Policy information about availability of computer code

Data collection	Sequencing data were obtained using Illumina sequencing device Hiseq2000.<br>Flow cytometry data were collected using DIVA<br>mIF images were collected using Panoramic P250 Flash III scanner (3DHistech) equipped with a Plan-Apochromat 20x/N.A. 0.8x objective (Carl Zeiss) and a Point Grey Grasshopper 5MP camera, using DAPI1, FITC, SpRed and Cy5 filter sets (Semrock)<br>Western blot images were obtained using FusionCaptAdvance solo4S (V16.2)<br>Microarray data were obtained with Affymetrix Genechips Operating Software
Data analysis	RNAseq data were analysed with: Hisat2(V2.0.5) ; StringTie(V1.3.3) ; STAR(2.5.3a); HTSeqCount; R(V3.5.2) ; GSEA(V3.0) ; DESeq2(V1.26.0)<br>Flow cytometry data were analysed with Flowjo (Treestar)<br>in-vivo experiments and statistical analysis were performed using JMP(V14); Excel(V16.21) or R(V3.5.2)<br>mIF images were analysed with: Halo<br>For Western blot quantification: Bio1D (V15.06b)<br>For microarray : Affymetrix Genechips Operating Software

For manuscripts utilizing custom algorithms or software that are central to the research but not yet described in published literature, software must be made available to editors/reviewers. We strongly encourage code deposition in a community repository (e.g. GitHub). See the Nature Research guidelines for submitting code & software for further information.

Data

Policy information about availability of data

All manuscripts must include a data availability statement. This statement should provide the following information, where applicable:
- Accession codes, unique identifiers, or web links for publicly available datasets
- A list of figures that have associated raw data
- A description of any restrictions on data availability

All data generated or analysed during this study are included in this published article (and its supplementary information files); on a public repository for RNAseq and Microarray data upon the accession codes : GSE153239, GSE153388, GSE154558 and GSE14330 ; or are available from the corresponding author on reasonable request.

Field-specific reporting

Please select the one below that is the best fit for your research. If you are not sure, read the appropriate sections before making your selection.

☒ Life sciences ☐ Behavioural & social sciences ☐ Ecological, evolutionary & environmental sciences

For a reference copy of the document with all sections, see nature.com/documents/nr-reporting-summary-flat.pdf

Life sciences study design

All studies must disclose on these points even when the disclosure is negative.

Sample size	No statistical methods were used to pre-determine sample size. Sample sizes were estimated based on preliminary experiments, which indicated that ±2 mice out of 10 would reject tumors when treated with anti-PD1 alone, whereas 4 to 5 mice out of 10 would reject tumors when treated with combination of anti-PD1 and anti-GARP. Thus, we estimated that we could not use group sizes inferior to 8-10 mice, without facing the risk of not observing rejection in the control group (anti-PD1 alone).
Data exclusions	No data were excluded, except in figure 5c and 5f/g in which 2 and 1 samples (out of a total of 40), respectively, were not analysed because cells were lost during flow cytometry.
Replication	Results shown in figure 2 were reproduced in ≥5 experiments. All attempt at replication were successful. A total of 4 and 3 independent experiments including similar groups were pooled in the meta-analyses shown in Figure 3a and 3b, respectively. Results shown in figures 4 and 6 were not reproduced in independent experiments, but differences were statistically significant for figure 6, and results were undisputable for figure 4 (complete rejection of CT26 in one flank but growth of 2 other tumors in all four responder mice) . Results shown in figure 5 were reproduced in 3 similar experiments, which are shown in supplementary figures. Experiments yielding RNaseq data shown in figure 4 and figure 8 were not reproduced independently, but contained a sufficient number of biological replicates to yield statistically significant robust interpretations.
Randomization	For all experiments shown in figure 2, 3, 5, 6 and 7 : before initiating mAb treatment, mice were randomly distributed into groups. Mean tumor surface, SEM, and proportions of females (F) and males (M) were calculated for each group and for the entire cohort of mice included in the study. Manual adjustments in grouping were made by swapping some mice between groups to ensure that tumor surface (mean and SEM) and proportions of F and M mice were equivalent between all groups.<br>For figure 1 there were no randomization as there were no replicates. For figure 4, all mice are derived from the same treatment group (responder mice) and therefore no randomization was applied.<br>For figure 8 no randomization was required because samples were not divided into multiple groups.
Blinding	No blinding was applied in our animal studies because these experiments must be run by a pre-designated scientist, to follow sanitary and ethical rules imposed by our local ethical committee and our animal facility manager.

Reporting for specific materials, systems and methods

We require information from authors about some types of materials, experimental systems and methods used in many studies. Here, indicate whether each material, system or method listed is relevant to your study. If you are not sure if a list item applies to your research, read the appropriate section before selecting a response.

Materials & experimental systems

n/a	Involved in the study
	☒ Antibodies
	☒ Eukaryotic cell lines
☒	Palaeontology
	☒ Animals and other organisms
	☒ Human research participants
☒	Clinical data

Methods

n/a	Involved in the study
☒	ChIP-seq
	☒ Flow cytometry
☒	MRI-based neuroimaging

Antibodies

Antibodies used	For in vivo-use : anti-mouseGARP:TGF-ß1 (clone 58A2, produced by co-authors from argenx); anti-humanGARP:TGF-ß1 (clones LHG10 and MHG8, produced in house and by co-authors from agenx); anti-PD1 FcS (clone RMP1-14, Absolute antibodies, Ab00813-2.3); anti-PD1 WT (clone RMP1-14, BioXcell, BE0146); anti-TGF-ß (clone 1D11, R&D System, BE0057); mIgG2a (motavizumab, evitria); anti-CD8 (clone 2.43, BioXcell,BE0061) ; anti-IFNg (clone FX4F3, see materials)<br>For FACS staining : anti-CD107a-BV421 (clone 1D4B, Biolegend, 121617) ; anti-CD16/CD32 (clone CI93, Biolegend,101320) ; anti-CD45-BV510 (clone 30-F11, Biolegend, 103138) ; anti-NKp46-BV786 (clone 29A1.4 , BD Pharmigen, 741029) ; anti-CD8a-PerCP (clone 53-6.7, Biolegend, BD Pharmigen) ; anti-CD4-FITC (clone GK1.5, Biolegend, 100406), anti-FOXP3-APC (clone FJK-16s, eBioscience, 17577382); anti-GARP-PE (clone YGIC86, ebioscience, 129891) ; anti-TNFa -BV711 (clone PM6-XT22, Biolegend, 506349); anti-IFNg-APC (clone XMG1.2, Biolegend, 505810); anti-HA (clone 16B12, Eurogentec, MMS-101R); Streptavidin-BV421 (BD Pharmigen, 563259); anti-LAP-APC (clone TW7-16B4 , Biolegend, 141406)<br>For Western Blot : anti-pSMAD2 (clone 138D4, cell Signaling Technology, 3108) ; anti-ß-actin (AC-15, Sigma, A5441)<br>For mIF : anti-GARP (MHG6, produced in house); anti-CD34 antibody (clone Qbend 10, Abcam, ab8536); anti-FOXP3 (clone D608R, Cell Signaling Technology, 12653) ; anti-CD3 (clone SP7, Abcam, ab16669) ; anti-CD8 (clone D4W2Z, Cell signaling, 98941)
Validation	- All commercially available antibodies used in-vivo, for flow cytometry, for western blot or for mIF were validated for their application by manufacturers.<br>- In house anti-GARP:TGFß clone 582 was developed for the purpose of the scietnific question addressed in this article. Results shown in the paper correspdond to the first validation of this antibody . Clone 58A2 binds GARP:TGF-ß1 complexes but not free GARP or free latent TGF-ß1 (Fig. 1a). It binds the surface of mouse Tregs, both resting and even more so after TCR stimulation (Fig. 1b). It also blocks the release of active TGF-ß1 induced by TCR stimulation of mouse Tregs in vitro (Fig. 1c), whether the mAb was used as a wild-type (WT) or an Fc-dead (FcD) mIgG2a subclass antibody. The FcD mIgG2a contains two amino-acid substitutions in the Fc region (D265A/N297A) that preclude binding to all mouse FcßRs22, 23 (Supplementary Figure 1). Binding and blocking activities of clone 58A2 closely resembled those of blocking anti-human GARP:TGF-ß1 mAbs.

Eukaryotic cell lines

Policy information about cell lines

Cell line source(s)	CT26 cell line was obtained from ATCC (CRL 2638)<br>EMT6 cell line was obtained from ATCC (CRL2755)<br>RENCA cell line was obtained from ATCC (CRL2947)<br>MC38 cell line was obtained from the laboratory of Dr Benoit van den Eynde (de Duve Institute, see refence doi: 10.1158/2326-6066.CIR-19-0041)<br>293 T cell line was obtained from ATCC (CRL3216)
Authentication	Cell lines from ATCC were authenticated by the ATCC, which provided a certificate of analysis (COA). Cells were not further authenticated in the lab.
Mycoplasma contamination	All cell lines were routinely tested for mycoplasma and were found to be negative.
Commonly misidentified lines<br>(See ICLAC register)	No commonly misidentified cell lines were used.

Animals and other organisms

Policy information about studies involving animals; ARRIVE guidelines recommended for reporting animal research

Laboratory animals	Males and females Balb/c and C57Bl/6 mice were bred from our institutional SPF platform (LAF) and were of age range from 6 to 13 weeks at the start of the experiments. The facility is controlled to maintain temperature between 20-24°C; HR between 40-65% and day-night cycles of 12h-12h.
Wild animals	The study did not involve wild animals
Field-collected samples	The study did not involve samples collected on the field

Ethics oversight	Animals experiments were in accordance with FELASA guidelines and were approved by the Ethical committee for Animal Experimentation of the "Secteur des Sciences de la Santé" of Université Catholique de Louvain under the reference 2015/UCL/MD/19 and 2019/UCL/MD/032

Note that full information on the approval of the study protocol must also be provided in the manuscript.

Human research participants

Policy information about studies involving human research participants

Population characteristics	The human cohort use in this study was made of metastatic melanoma patients (skin and eye) including 11 females and 6 males of age ranging from 29 to 83 years old. Tumor material was obtain after resection of skin metastasis.
Recruitment	We have used archived cryopreserved tumor samples from a biobank collected between 2010 and 2013 for other purposes than the present study. The samples were selected based on the following criteria: (i) cutaneous metastasis from skin melanoma, (ii) quality of the tissue and presence of tumor areas based on an hematoxylin-eosin stained section and (iii) quality of total RNA extracted. Twenty samples were chosen. One sample showed non interpretable immunostaining results and was excluded (not provided here). One tumor sample was found retrospectively to have originated from an ocular melanoma and was kept.
Ethics oversight	Tumors and healthy tissues were obtained as surgical discard samples, or as research-aimed surgery or biopsy, after informed consent and under approval of the Commission d'Ethique Biomédicale Hospitalo-Facultaire, Brussels, Belgium (reference CEHF 2014/457).

Note that full information on the approval of the study protocol must also be provided in the manuscript.

Flow Cytometry

Plots

Confirm that:

- [x] The axis labels state the marker and fluorochrome used (e.g. CD4-FITC).
- [x] The axis scales are clearly visible. Include numbers along axes only for bottom left plot of group (a 'group' is an analysis of identical markers).
- [x] All plots are contour plots with outliers or pseudocolor plots.
- [x] A numerical value for number of cells or percentage (with statistics) is provided.

Methodology

Sample preparation	Tumors were collected and cut into 2 fragments of approximately 2/3 and 1/3 of the the original size. The largest fragments were placed in wells of a 6-well plate containing 5ml Iscove's modified Dulbeco's Medium and enzymes (Collagenase I 100mg/ml, Life Tech; Collagenase II 100mg/ml, Life Tech; Dispase 1mg/ml ,Life Tech; and DNAse I 0.4U/ml, Roche). Fragments were cut into small pieces with surgical scalpel blades then transfer in GentleMACS C-tube for two further mechanical dissociation cycles interrupted by an incubation of 30min at 37°C under continuous rotation.<br>Cell suspensions were pass through 70µm cell strainers and centrifuged. Supernatant was discarded and cell pellets were resuspended in 10ml FACS buffer before passing through 40µm strainers and counting on a Luna device.<br>Cells were put at a concentration of 10^7 cells/ml and 100µl of the suspension was distributed in adequate 96 FACS plate.<br>After 4h of stimulation in adequate mix, cells were centrifuged and supernatant was removed by splashing the plates. Surface staining mix was added for 20min on ice in the dark. Cells were then washed 2x in FACS buffer and resuspended in fixation/permeabilization buffer for 20min (for cytokines) or overnight (for transcription factors). Cells were washed and resuspended in FCγR blocking mix for 10min on ice before 20min (for cytokines) or 1h (for transcription factors) of incubation with intracellular staining mixes. Finally, cells were washed and resuspended into FACS buffer.
Instrument	FACS LSR Fortessa
Software	Data collection was performed usind DIVA and analysis with FlowJo
Cell population abundance	No pre-sorting was performed on the samples
Gating strategy	See Supplementary Fig. 13 for gating strategy

- [x] Tick this box to confirm that a figure exemplifying the gating strategy is provided in the Supplementary Information.
